# GKNnet: an relational graph convolutional network-based method with knowledge-augmented activation layer for microbial structural variation detection

**DOI:** 10.1093/bib/bbaf200

**Published:** 2025-05-05

**Authors:** Fengyi Guo, Yuanbo Li, Hongyuan Zhao, Xiaogang Liu, Jian Mao, Dongna Ma, Shuangping Liu

**Affiliations:** School of Artificial Intelligence and Computer Science, Jiangnan University, 1800 Lihu Avenue, Binhu District, Wuxi, Jiangsu 214122, China; School of Artificial Intelligence and Computer Science, Jiangnan University, 1800 Lihu Avenue, Binhu District, Wuxi, Jiangsu 214122, China; National Engineering Research Center of Cereal Fermentation and Food Biomanufacturing, State Key Laboratory of Food Science and Technology, School of Food Science and Technology, Jiangnan University, 1800 Lihu Avenue, Binhu District, Wuxi, Jiangsu 214122, China; Luzhou Laojiao Group Co. Ltd, 157 Guojiao Road, Jiangyang District, Luzhou 646000, Sichuan, China; National Engineering Research Center of Cereal Fermentation and Food Biomanufacturing, State Key Laboratory of Food Science and Technology, School of Food Science and Technology, Jiangnan University, 1800 Lihu Avenue, Binhu District, Wuxi, Jiangsu 214122, China; Shaoxing Key Laboratory of Traditional Fermentation Food and Human Health, Jiangnan University (Shaoxing) Industrial Technology Research Institute, Keqiao District, Shaoxing 312000, Zhejiang, China; National Engineering Research Center of Cereal Fermentation and Food Biomanufacturing, State Key Laboratory of Food Science and Technology, School of Food Science and Technology, Jiangnan University, 1800 Lihu Avenue, Binhu District, Wuxi, Jiangsu 214122, China; School of Artificial Intelligence and Computer Science, Jiangnan University, 1800 Lihu Avenue, Binhu District, Wuxi, Jiangsu 214122, China; National Engineering Research Center of Cereal Fermentation and Food Biomanufacturing, State Key Laboratory of Food Science and Technology, School of Food Science and Technology, Jiangnan University, 1800 Lihu Avenue, Binhu District, Wuxi, Jiangsu 214122, China; Luzhou Laojiao Group Co. Ltd, 157 Guojiao Road, Jiangyang District, Luzhou 646000, Sichuan, China

**Keywords:** structural variation detection, long-read sequencing, heterogeneous graph, graph neural network

## Abstract

Structural variants (SVs) in microbial genomes play a critical role in phenotypic changes, environmental adaptation, and species evolution, with deletion variations particularly closely linked to phenotypic traits. Therefore, accurate and comprehensive identification of deletion variations is essential. Although long-read sequencing technology can detect more SVs, its high error rate introduces substantial noise, leading to high false-positive and low recall rates in existing SV detection algorithms. This paper presents an SV detection method based on graph convolutional networks (GCNs). The model first represents node features through a heterogeneous graph, leveraging the GCN to precisely identify variant regions. Additionally, a knowledge-augmented activation layer (KANLayer) with a learnable activation function is introduced to reduce noise around variant regions, thereby improving model precision and reducing false positives. A clustering algorithm then aggregates multiple overlapping regions near the variant center into a single accurate SV interval, further enhancing recall. Validation on both simulated and real datasets demonstrates that our method achieves superior F1 scores compared to benchmark methods (cuteSV, Sniffles, Svim, and Pbsv), highlighting its advantage and robustness in SV detection and offering an innovative solution for microbial genome structural variation research.

## Introduction

Genomic structural variants (SVs) refer to structural alterations in chromosomes or large DNA segments that are typically over 50 bp in length. These variants usually include forms such as insertions, deletions, duplications, translocations, and inversions [1]. Research has shown that SVs are extensively involved in biological phenotypic variation, environmental adaptation, and speciation [2]. As an important model organism in biomedical research, *Saccharomyces cerevisiae* (brewer's yeast) is widely applied in various industries, including food, energy, and pharmaceuticals [3]. The continuous accumulation of structural variants in the genome of *S. cerevisiae* drives the gradual optimization of its phenotypes, allowing it to adapt to changing environments [4]. Techniques such as gene amplification and targeted mutagenesis can enhance yeast's tolerance, fermentation efficiency, and drug resistance [5]. Accurately identifying key SVs associated with phenotypes would facilitate functional modification of specific regions in the yeast genome, significantly boosting its potential for industrial applications.

Compared to short-read sequencing, long-read sequencing offers unique advantages, especially in spanning highly repetitive regions and long-range genomic regions. This capability significantly improves alignment quality and enables the detection of a greater number of SVs [6, 7]. However, while long-read sequencing improves alignment and detection sensitivity, it also introduces considerable noise, leading to increased false-positive and reduced recall rates. These high false-positive and low recall rates are prevalent issues in current long-read sequencing technologies, limiting their precision in SV detection. Missed key SVs may have profound impacts on biological phenotypic changes, a challenge that also affects SV detection in *S. cerevisiae*. Therefore, effectively enhancing the accuracy and sensitivity of SV detection in *S. cerevisiae* is not only a critical scientific question for uncovering its environmental adaptability but also has potential implications for increasing its value in industrial applications [8].

Currently, mainstream methods for detecting SVs are mainly divided into two major categories [9]: One approach is based on *de novo* assembly, while the other relies on read alignment. The *de novo* assembly strategy does not depend on a reference genome; instead, it directly assembles and aligns sequencing reads, and it is widely used in genomic analysis. This method can detect large-scale structural variants and construct more complete genomic maps, making it particularly advantageous for handling complex SVs. However, the *de novo* assembly process requires sequence preprocessing to remove redundancies, and when dealing with plant or complex eukaryotic genomes, the abundance of repetitive sequences can lead to assembly errors [10, 11]. In addition, this strategy is time-consuming and requires high computational resources, limiting its efficiency in large-scale applications. In contrast, the read alignment-based strategy aligns long-read sequencing fragments to a reference genome, locating each read's position in the original genome to identify and detect SVs [12]. Currently, minimap2 [13] and BWA [14] are widely used and efficient alignment tools. Many SV detection workflows first use alignment tools to obtain alignment information for sequencing data and then combine specific strategies or clustering methods to accurately interpret SVs. The read alignment-based strategy typically achieves fast and accurate alignment by constructing an index of the reference genome.

In recent years, deep learning methods have made significant progress in the field of SV detection [15]. By transforming sequence information into images, as in DeepSV [16], or feature matrices, as in Breaknet [17], these models can automatically learn sequence features to detect SVs. However, these methods have certain limitations: Since the length of long reads is typically around 10 000 bp, directly converting the entire read information into images or matrices for model input incurs a substantial computational cost. Therefore, sliding window techniques are often employed to divide the images or matrices into smaller windows, standardizing their size for model training. In addition, for larger SVs (several thousand bps), complete detection becomes challenging, as feature information may be fragmented and lost. To address these challenges, graph convolutional networks (GCNs) have demonstrated unique advantages in SV detection. GCNs are trained directly on graph-based, unstructured data, effectively reducing feature loss. Their local information aggregation mechanism allows the model to capture contextual information between the nodes. For example, in the regions adjacent to deletions, sequence disruptions often occur, and GCN can more effectively identify these features. Additionally, even when the entire long read is constructed into a graph, the memory requirements for GCN remain relatively low. Its robustness allows it to maintain good performance even in high-noise data. GCNs also offer excellent scalability and adaptability, making them suitable for graph data of varying sizes [18]. Through the convolution operation, GCN dynamically constructs feature representations and adaptively adjusts them based on the structure of the graph.

In this study, we propose a new model, GKNnet, which applies GCN to detect deletion variants in *S. cerevisiae*. The model demonstrates outstanding performance on both simulated and real datasets, particularly showing a significant advantage on low-coverage datasets.

## Materials and Methods

### Data

This study used the S288C genome from the *S. cerevisiae* reference genome ScRAP [19], assembled with the telomere-to-telomere approach, as a reference. Based on this reference genome, simulated datasets were generated using the sim-it software [20]. Additionally, two well-studied datasets, ERR8562466 and DRR095880, were utilized in the experiments, originating from two different sequencing platforms—Oxford Nanopore and PacBio. By incorporating sequencing data from diverse platforms, this study validated the stability and accuracy of the model under varied data conditions.

### Methods

**Figure 1:**
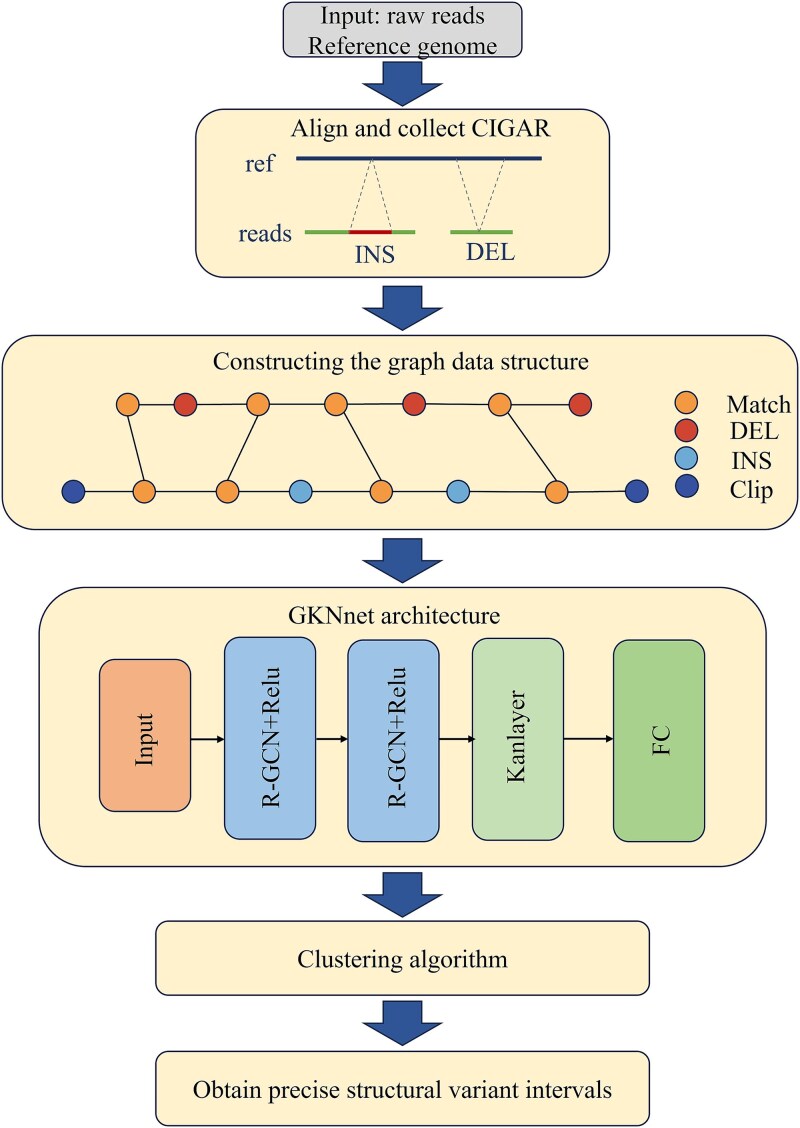
The data flow diagram. (i) Alignment information extraction: Align sequencing data to the reference genome to obtain CIGAR information. (ii) Representation information: Convert CIGAR into a graph data structure through a graph construction strategy. (iii) Node classification: Use the GKNnet model to identify variant nodes. (iv) Node clustering: Apply a new clustering strategy to obtain accurate SVs.

In this study, we developed a model based on GCN and long-read sequencing technology to detect deletion variants. This model involves three key steps, as illustrated in Fig. 1:

(i) Alignment and graph data transformation: First, sequencing reads are aligned to the reference genome, and alignment information is converted into a graph structure using heterogeneous graph data features [21].

(ii) Candidate variant detection: A GCN is used to perform node classification within the graph to identify potential variant regions. Leveraging GCN’s local information aggregation mechanism, the model captures local contextual information in the sequence, enabling more accurate detection of candidate variant regions.

(iii) Precise classification of candidate regions: Finally, a clustering algorithm is applied to refine the classification of candidate variant regions, further enhancing detection accuracy and yielding the final variant intervals.

### Building graph data structures

We used the minimap2 alignment tool to align sequencing reads to the reference genome and generated BAM files containing alignment information for each read. In the data preprocessing stage, the reads were filtered based on FLAG values: Reads with FLAG values of four or greater than or equal to 256 were removed. Additionally, reads with variant lengths of less than 50 bp were excluded [22]. The filtered reads were then constructed into a graph data structure, as shown in Fig. 1.

The main types of nodes in the graph include match, insertion, deletion, and clipping, labeled as 0, 1, 2, and 3, respectively. To minimize the impact of complex translocations and inversions on other variant types, these complex variants were excluded. Next, we generated the graph based on Compact Idiosyncratic Gapped Alignment Report (CIGAR) string information, with two types of nodes: reference genome sequence nodes and sequencing read nodes. Each node's features were represented by six dimensions: (*v*1, *v*2, *v*3, *v*4, start, and end). Here, *v*1 to *v*4 correspond to the four-node types, with the dimension for the node’s specific type reflecting the variant length. The *start* and *end* dimensions represent the starting and ending positions of the node in the sequencing read.

Heterogeneous graphs have demonstrated superior performance across various fields, such as social networks, gene regulatory networks, and more [23, 24]. In this study, graph relationships were categorized into three types: ref-ref, ref-read, and read-read. Two relationship modeling approaches were tested in our experiments. The first approach consolidated all nodes and edges into a single adjacency matrix, with different relationships distinguished by edge weights. The second approach created a separate adjacency matrix for each relationship type, forming a multi-relational adjacency matrix stored as a sparse matrix to reduce storage occupied by zero elements. To improve feature extraction efficiency, the model assigned different weights to adjacency matrices for each relationship, allowing for more precise learning of structural features within the graph. Although the multi-adjacency matrix approach incurs a slight increase in computational overhead compared to a single matrix, it provides clearer and more accurate feature extraction. Furthermore, this multi-channel design enhances data dimensionality, offering richer information for model training. Therefore, the multi-relational matrix approach was adopted for constructing the graph’s adjacency matrix in subsequent experiments.

### Detect candidate deletions

In this step, the model utilizes relational GCN [21] convolutional layers for graph convolution operations. By extending traditional convolutional neural networks (CNNs) to graph data structures, GCN achieves robust classification performance. This capability is especially valuable in bioinformatics, where complex node types and relationships present significant challenges. The architecture of our model is shown in Fig. 1, illustrating how GCN demonstrates powerful classification performance in handling these complexities.

In microbial genome data, structural variation (SV) intervals are much fewer than normal intervals. Traditional CNNs often require converting data into image or matrix formats, which involve normalization that may split complete SVs, making them difficult to reassemble. This affects model accuracy and performance. In contrast, graph data structures solve this issue by representing each read as a graph, which captures global features and avoids feature loss due to splitting.

In this study, we use a heterogeneous graph structure, which is especially effective for microbial genome data. Heterogeneous graphs use different node and edge types, representing complex relationships, such as those between reference genome nodes and sequencing read nodes. This structure allows for better representation of variant regions without losing important features. GCNs based on this structure effectively capture complex relationships between the nodes. GCNs aggregate local information by dynamically adjusting feature representations based on graph structure, avoiding feature loss common in traditional methods. GCNs handle unstructured data efficiently and capture contextual information, particularly in SV regions, improving variant feature extraction.

Microbial genome data also contain significant noise, especially in low-coverage data. To address this, we introduced the knowledge-augmented activation layer (KANLayer) module. With its learnable activation function, KANLayer adapts to varying data distributions and suppresses noise, enhancing the model’s ability to extract relevant SV features. By combining GCN and KANLayer, the model improves SV detection accuracy and stability. GCN captures global features from long-read sequences, while KANLayer reduces noise and enhances precision. This combination optimizes the SV detection process, ensuring high performance in complex, noisy data typical of microbial genomes.

The model’s input size is (*N*,6), where *N* represents the number of nodes, and each node has a feature dimension of 6. We built the GKNnet model using two graph convolution layers. For the three different types of relationships, we utilized three adjacency matrices, assigning each adjacency matrix a distinct weight matrix for learning. Between the convolution and fully connected layers, we introduced KANLayer [25] to enhance the model’s representation of nodes and their relationships. Equipped with a learnable activation function, KANLayer can dynamically adjust activation thresholds based on input data, giving the model increased flexibility and adaptability in feature learning. This dynamic adjustment mechanism not only helps the model adapt to varying feature distributions but also effectively reduces noise interference, resulting in more precise feature extraction. From the above description, it can be seen that our model is lightweight, with only 5.08 × 10^−4^ M parameters. For computational complexity, we use an average-sized graph for evaluation, which contains 1 k nodes and 500 edges, resulting in a computation cost of 114 000 FLOPs. The detailed model architecture and parameters are found in Supplementary Table S1.

Additionally, KANLayer optimizes relationship representation among nodes in complex graph structures, further strengthening the model’s classification and detection capabilities within a complex GCN architecture. By incorporating KANLayer, the model achieves significantly improved precision while maintaining high recall, ensuring stable and reliable results.

For the loss function, cross-entropy loss is used to calculate the loss, with the label having the highest probability chosen as the output for each node. The graph convolution formula employed in our model is as follows:


(1) \begin{equation*} {H_i}^{\left(l+1\right)}=\sigma \left[\sum \limits_{\mathrm{r}\in R}\sum \limits_{\mathrm{j}\in{N}_i^r}\frac{1}{C_{i,r}}{W_r}^{(l)}{H}_j^{(l)}+{W_0}^{(l)}{H}_i^{(l)}\right] \end{equation*}

Here, ${H}_i^{\left(l+1\right)}$ represents the embedding of node *i* at the *l* + 1 layer. R is the set of all relationships, ${N}_i^r$ represents the neighbors of node *i* connected through relationship *r*,${W}_r^l$ is the trainable weight matrix corresponding to relationship *r*, ${C}_{i,r}$ is the normalization factor for relationship *r*, which incorporates node degree information, $\sigma$is the non-linear activation function, and in this study, we used the ReLU activation function.

The formula for the loss function used in our model is as follows:


(2) \begin{equation*} Loss=-\frac{1}{N}\sum \limits_{i=1}^N{w}_{y_i}\log \left({p}_{i,{y}_i}\right)+\frac{\lambda }{2}{\left\Vert W\right\Vert}_2^2 \end{equation*}

Here, ${w}_{y_i}$is the weighted cross-entropy loss, adjusted according to the class weights [1-weight, weight], ${p}_{i,{y}_i}$ is the model's predicted probability for the true class ${y}_i$ of sample *i*. The second term is the L2 regularization, which is used to constrain the model parameters *W* and prevent overfitting.

### Processing of candidate SV regions

In our study, we first extracted candidate deletion variant region nodes using deep learning methods. For the same variant region, multiple long-read alignments may exist, but each alignment may have slight discrepancies in its start and end positions. These discrepancies are primarily caused by sequencing errors, limitations of alignment algorithms, and sequence complexity. To eliminate these errors and obtain accurate variant region centers, we applied clustering algorithms from machine learning. In the clustering process, we first grouped the data based on chromosome numbers and then performed clustering by calculating the Euclidean distance of each node's variant length, start position, and end position. This process treats each node as a data point; calculates the distance in terms of variant length, start position, and end position; and assigns the node to the corresponding cluster, effectively grouping the nodes within the same variant region. We chose a density-based clustering algorithm because it does not require pre-setting the number of clusters and is well-suited for our research scenario. This algorithm allows us to avoid the issues of complex clustering methods that may lead to confusing results and slow computation speeds and eliminates the need to determine the number of clusters before detection is completed. However, after the initial clustering, we found that when dealing with longer deletion variant regions, the algorithm struggled to accurately identify the entire variant region and often split it into several adjacent variant region fragments. To address this issue, we designed Formula 3 to handle the regions not matched in the first clustering. When these regions meet any condition in the formula, we merge them to obtain more accurate deletion variant regions. Testing the model on the simulated dataset showed that after using the clustering algorithm, the accuracy increased significantly by 15%, and the recall rate improved by 5%. This improvement significantly enhanced the precision of variant detection and effectively reduced the impact of sequencing errors and alignment biases. Therefore, the use of a density-based clustering algorithm played a crucial role in enhancing the accuracy of variant region identification.


(3) \begin{equation*} \left\{\begin{array}{l} start2- end1<\alpha \\{}\frac{\min \left( svlen1, svlen2\right)}{\max \left( svlen1, svlen2\right)}>0.7\end{array}\right. \end{equation*}

Here, *start* represents the starting position of the deletion variant region, *end* represents the ending position of the deletion variant region, and “svlen” represents the variant length of the region. The value of *α* is selected based on the specific sequencing data to set an appropriate threshold for aggregation, and in this study, it is set to 3000. Using Formula 3, nodes that were not mapped to the accurate region are aggregated, followed by secondary clustering. This clustering strategy effectively addresses the issue of large alignment errors for long variant regions in the model.

## Results and Discussion

### Evaluation results of different variant callers on simulated data

**Figure 2:**
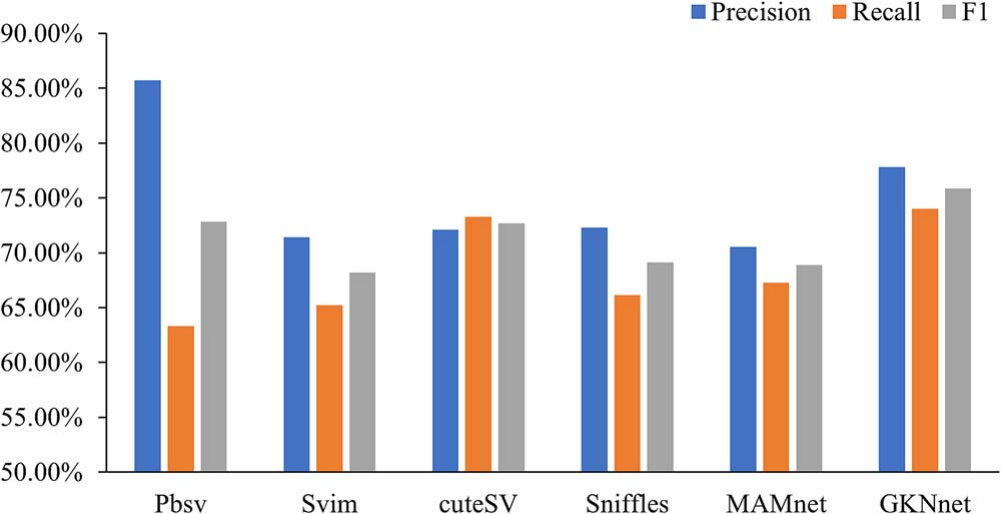
Performance comparison on the simulated dataset.

We employed four structural variation detection methods: Pbsv, Svim, cuteSV, and Sniffles, with minimap2 and pbmm2 alignment tools for comparison. First, we trained the model using simulated data from chromosomes I to VI, and the remaining part was used for model validation. The simulated data were generated using the sim-it software and based on the S288C reference genome. Figure 2 (Supplementary Table S2) displays the benchmarking results and performance comparison of the model on the simulated dataset. The results show that in terms of recall and F1 score—two key metrics—our model outperforms the other four methods. Although our model slightly lags behind Pbsv in precision, it is noteworthy that Pbsv has a 10% lower recall rate than our model. The excellent performance of our model is primarily attributed to the KANLayer architecture, which effectively extracts data features during multiple convolutional iterations, successfully attenuating noise near the variant regions. Compared to the LSTM-based MAMnet model, the overall performance of GKNnet shows improvements, particularly in recall and F1 score, where our model outperforms MAMnet. As a result, our model demonstrates a significant improvement in recall for SV detection. To validate the generalizability of our model, we used two real strain sequencing datasets, ERR8562466 and DRR095880. These datasets were obtained from the Oxford Nanopore and PacBio sequencing platforms, respectively. ERR8562466 has an initial sequencing depth of 210X, with random samplings of 168X, 126X, and 50X data; DRR095880 has an initial depth of 250X, with random samplings of 125X, 60X, and 30X. Due to the lack of a high-confidence structural variation benchmark set for *S. cerevisiae*, we merged the VCF files generated by other baseline methods using combiSV to form a reference set for subsequent comparative analysis. During the experimental process, we found that the cuteSV method failed to provide any valid results each time it processed the data and was incompatible with the other three methods. Therefore, we ultimately decided to use the remaining three baseline methods for testing and comparison.

This section conducts a comparative experiment to demonstrate the performance enhancement brought by the KANLayer module to the model. Its learnable activation function facilitates the model's ability to capture global features. Initially, KANLayer was designed as an alternative to Multilayer Perceptron (MLP) for classification tasks. Various configurations, including FC, KANLayer, two-layer KANLayer, and KANLayer + FC, were tested on the simulated dataset. Given the lower feature dimensionality in the model, we opted for a single-layer FC and did not explore deeper multi-layer FC architectures. As shown in Table 1, the experimental results indicate that a model with a single KANLayer performs worse than a single-layer FC. Employing a two-layer KANLayer significantly led to overfitting, misclassifying variant nodes as normal ones. Ultimately, we found that combining KANLayer with FC improved the model's ability to learn global features while enhancing classification performance, accurately identifying variant nodes.

**Table 1:** The evaluation of KANLayer and fully connected layers in variant detection

	FC	Kanlayer	Kanlayer^*^2	Kanlayer + FC
Precision	70.21%	67.26%	56.37%	77.81%
Recall	68.16%	63.28%	51.69%	74.01%
F1	69.17%	65.21%	53.93%	75.86%

### Evaluation results of ERR8562466 from different variant callers based on Oxford nanopore sequencing data

**Figure 3:**
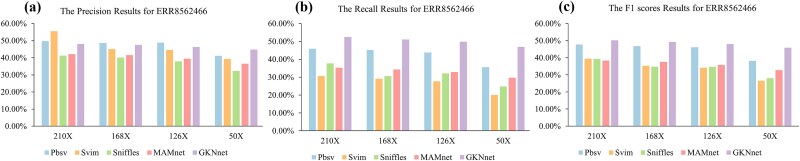
(a) Precision results for deletion detection on the ERR8562466 dataset. (b) Recall results for deletion detection on the ERR8562466 dataset. (c) F1 scores results for deletion detection on the ERR8562466 dataset.

We used the chromosome 9–16 dataset as a validation set for both the model and the baseline methods, with the benchmarking results shown in Fig. 3 (Supplementary Table S3). The results indicate that, at a coverage depth of 210X, although the precision of our model is not the highest—just 7% lower than the optimal Svim—it achieves a 22% higher recall compared to Svim. As seen in the table, the model shows a significant improvement in recall compared to the baseline methods and performs best in terms of the F1 score. This demonstrates that, in high-coverage datasets, the model performs well. To evaluate the impact of coverage depth on model performance, we randomly sampled data at different depths using samtools. At a coverage depth of 168X, the model’s accuracy improved to the second-best result, with minimal impact on both the model and the Pbsv method but significant effects on the Svim and Sniffles methods. In terms of recall and F1 score, the model still outperformed all other methods. At a coverage depth of 126X, the model continued to outperform the baseline methods in recall and F1, with only minor changes compared to the 168X results. This may be due to the relatively small size of the *S. cerevisiae* genome and the low number of SVs, meaning that slight reductions in coverage depth did not cause significant changes.

Next, we randomly extracted data at 50X coverage depth to observe the impact of coverage depth on detection performance. At this lower coverage, all three baseline methods experienced a significant performance loss. Although precision was slightly affected, recall dropped by approximately 10%. In contrast, the model showed minimal impact on performance, maintaining superior recall and F1 score and achieving the best precision, which was approximately 3% higher than Pbsv. This suggests that the model performs excellently at different coverage depths.

### Evaluation results of DRR095880 from different variant callers based on PacBio sequencing data

**Figure 4:**
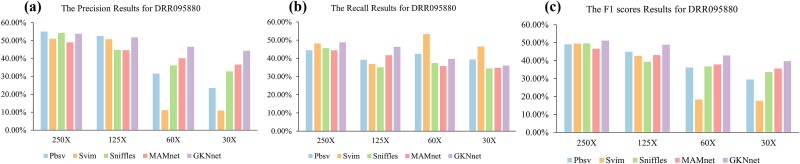
(a) Precision results for deletion detection on the DRR095880 dataset. (b) Recall results for deletion detection on the DRR095880 dataset. (c) F1 score results for deletion detection on the DRR095880 dataset.

We used the chromosome 9–16 dataset as a validation set for both the model and the baseline methods, with the benchmarking results presented in Fig. 4 (Supplementary Table S4). At a coverage depth of 250X, although the model's precision was not the highest, it was only 1% lower than Pbsv, which performed best in precision. In terms of recall, the model still showed excellent performance, outperforming all other baseline methods. While the recall of Svim was close to that of the model, its precision decreased by 3%. In the F1 score, the model continued to perform the best, outperforming the other baseline methods. In the downsampling experiment on ERR8562466, we observed that reducing the coverage depth at ratios of 0.8 and 0.6 had minimal impact on performance. Therefore, in subsequent experiments, we adopted a strategy of halving the coverage depth at each step. At a coverage depth of 125X, the recall rate of the baseline methods was significantly affected, with Svim and Sniffles experiencing a 10% decrease and Pbsv showing a 5% decrease, while the model’s performance loss was relatively small, with losses of around 2% for all three metrics. The model still performed the best in recall and F1 score. At a coverage depth of 60X, all baseline methods showed a notable decline in performance, particularly Svim, where the reduction in coverage depth exacerbated the false-positive rate. Pbsv and Sniffles showed a similar trend, with a significant drop in precision. In contrast, while the model also experienced some performance loss at this lower coverage depth, its performance across all three metrics remained the best, suggesting that the model is less affected by lower coverage depth. At a coverage depth of 30X, the impact of coverage depth on both the model and the baseline methods became more pronounced. As the coverage depth further decreased, the performance of all methods declined significantly. In terms of precision, the model performed the best; although the recall was 10% lower than the optimal Svim, Svim's precision was much lower than that of the model. In terms of F1 score, the model still outperformed all other methods.

From the two experiments above, it is evident that, although the model's precision does not always achieve the highest performance across different sequencing platforms and depths, it consistently delivers the best results in terms of recall and F1 score. While ONT data have longer read lengths, they have lower base accuracy and more sequencing errors compared to PacBio data, which offer higher accuracy. Additionally, ONT data often require extra post-processing and data correction. As a result, PacBio sequencing data hold a clear advantage in structural variation analysis. However, the model also demonstrated strong performance on ONT data, indicating its strong generalization ability across different sequencing platforms. Regarding different sequencing depths, the model also exhibited excellent generalization. Next, we will test the model's sensitivity to SVs of different lengths. Due to the relatively small *S. cerevisiae* genome and the lack of accurate, high-confidence datasets, we used simulated datasets for experiments with varying SV lengths. The advantage of using simulated datasets is that they provide a relatively precise training set, allowing for better analysis. These controllable datasets more accurately reflect the model's capability in detecting SVs of different lengths.

### The performance for deletion with different lengths on simulated data

**Figure 5:**
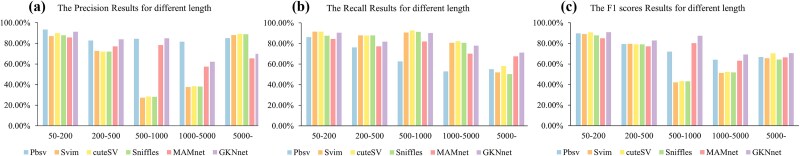
(a) Precision results of method experiments on varying length groups in simulated dataset. (b) Recall results of method experiments on varying length groups in simulated dataset. (c) F1 score results of method experiments on varying length groups in a simulated dataset.

To evaluate the model's performance in detecting SVs of different lengths, we divided the interval lengths into five categories: [50, 200], [200, 500], [500, 1000], [1000, 5000], and [5000, ∞]. The benchmarking results for different lengths in the simulated dataset are shown in Fig. 5 (Supplementary Table S5). The model achieved the best F1 score in each length interval. In the [50, 200] interval, both the baseline methods and the model showed good performance. Although the model's precision and recall did not reach the optimal values, the differences from the best baseline method were minimal, allowing the model to achieve the best F1 score. In the [200, 500] interval, the model achieved the highest precision. Although the recall was 6% lower than that of the optimal baseline method, the increase in precision allowed the model to still achieve the best F1 score. In the [500, 1000] interval, Svim, cuteSV, and Sniffles detected more SVs, with recall exceeding 90%, but they suffered from significant false positives. The model had the best precision, and its recall was only 2% lower than the optimal cuteSV. Its F1 score was 40% higher than all methods except Pbsv. In the [1000, 5000] interval, the model's precision was second only to the optimal Pbsv, but the model detected more SVs, and recall was good. Although not the highest, the model achieved the best overall F1 score. In the [5000, ∞] interval, the baseline methods could only detect a few SVs, while the model’s recall reached 71%, surpassing the average recall of the baseline methods by 20% and achieving the best performance in terms of F1 score. For larger length intervals, despite a slight decrease in precision, the model's recall was outstanding, addressing the issues of low detection and high false positives for large-length variants.

The experimental results indicate that the model performs exceptionally well on long-read sequencing data, especially in low-coverage datasets. From the above results, it can be seen that GKNnet performs well and shows an improvement in performance compared to MAMnet. The poor performance of the MAMnet model on microbial genomes is primarily due to the format of its feature matrix, which causes some loss of global features when translating the characteristics of microbial sequencing data, resulting in suboptimal performance. Leveraging the iterative feature extraction capability of KANLayer and the advantages of the graph data format features, the model maintains stability and minimizes performance loss as coverage depth decreases. Through a novel clustering strategy, the model significantly enhances precision and recall in detecting larger SVs, effectively stitching fragmented variants caused by alignment tool errors into complete and accurate SVs. Typically, long-read sequencing data require high-coverage depths to achieve optimal SV detection performance, which substantially increases costs. However, the outstanding performance of our model on low-coverage datasets makes it a more cost-effective choice [9]. We further validated the good generalization ability of our model on other microbial species, which was thoroughly demonstrated through experiments on two datasets: Saccharomyces cerevisiae and Penicillium species. The specific experimental results can be found in the supplementary materials S6 and S7.

Although this study has achieved certain results, there are still some limitations that provide potential areas for future improvement. GKNnet can only detect deletion variants, while other types of structural variants, such as insertions and duplications, remain unaddressed. This limitation primarily stems from the current graph construction strategy of GKNnet, which does not effectively represent the characteristics of these structural variants. To detect other types of structural variants, the graph construction strategy needs to be modified to better accommodate their data characteristics. For example, when detecting deletion variants, GKNnet mainly learns feature data from the reference genome graph, which contains only two types of nodes: normal nodes and deletion variant nodes. However, insertions and other structural variants are distributed along sequencing reads. Therefore, additional relational edges need to be incorporated into the graph to enhance the learning of insertion variant nodes and the interaction with their neighboring nodes.

Furthermore, GKNnet has efficiency limitations (for detailed information, see Supplementary Material S8). Although it outperforms baseline methods in terms of accuracy, efficiency remains a concern. In future research, we plan to design a pruning and quantization strategy to reduce computational burden by removing nodes that have minimal impact on structural variant detection. Currently, GKNnet uses float32 for computation, but reducing the precision to float16 or INT8 could decrease memory consumption and accelerate matrix operations. However, quantization may affect accuracy, which requires further investigation in future studies.

## Conclusion

This paper presents GKNnet, a deletion-type structural variation (SV) detection method that integrates GCNs with long-read sequencing technology. By constructing a heterogeneous graph-based data structure, GKNnet enhances the expression of variant relationships, leading to improved detection performance. Experimental results demonstrate that the model shows a clear performance advantage over benchmark methods. Notably, GKNnet maintains greater stability under low-coverage conditions, making it suitable for low-coverage SV detection in practical applications. Furthermore, GKNnet's adaptability across different sequencing platforms broadens its applicability in various microbial detection contexts. Overall, while GKNnet does not consistently outperform in every detection metric, its exceptional performance in low-coverage and cross-platform adaptability indicates strong advantages for real-world use.

However, GKNnet does have limitations, excelling primarily in detecting deletion variants. For more complex variant types, such as inversions and translocations, the model's performance is comparatively limited. This is mainly due to the existing graph structure's limitations in accurately representing inversion and inter-chromosomal translocation relationships. Additionally, the current clustering strategy is confined to clustering variations within a single chromosome. Adapting GKNnet to detect other SV types would require appropriate adjustments in graph construction and clustering strategies to address these more complex variations.

**Key Points**

- Microbial phenotypic changes, environmental adaptation, and speciation are strongly linked to genomic Structural Variants, with a high correlation observed between deletion variants and these phenotypic traits.
- Long-read sequencing data can identify more Structural Variants (SVs) and significantly enhance the performance of detection algorithms.
- Using a heterogeneous graph to construct the graph data structure fully represents the relationships between node variants.
- Using both simulated and real datasets, we show that our model, GKNnet, surpasses Pbsv, Svim, Sniffles, and cuteSV in F1 score, demonstrating enhanced detection accuracy.

## Supplementary Material

supplymentary_data_bbaf200

## Data Availability

The additional data and code related to this paper can be downloaded from https://github.com/GuoFY-nn/GKNnet.
