# Supplementary material for: GKNnet: an relational graph convolutional network-based method with knowledge-augmented activation layer for microbial structural variation detection
Source: Brief Bioinform. 2025 May 5;26(3):bbaf200. doi: 10.1093/bib/bbaf200 (PMC12052243; doi:10.1093/bib/bbaf200)
Supplement: supplymentary_data_bbaf200 [file supplymentary_data_bbaf200.docx]

**Table S1.** Model Architecture and Parameter Statistics Table

| Layer Name | Input Dimension (*d_in_*) | Output Dimension (*d_out_*) | Number of Relations (*r*) | Number of Parameters |
| --- | --- | --- | --- | --- |
| R-GCN Layer 1 | 6 | 12 | 3 | 228 |
| R-GCN Layer 2 | 12 | 6 | 3 | 222 |
| Kanlayer | 6 | 6 | - | 44 |
| Fully Connected | 6 | 2 | - | 14 |
| Total | - | - | - | 508 |

Computational Complexity Analysis:

The computational complexity for each layer of the model is as follows:

R-GCN Layer 1 and Layer 2:

The computational complexity for each layer is ,where$\left|E\right|$represents the number of edges, $\left|V\right|$represents the number of nodes, and d is the node embedding dimension. 在In Layer 1, din=6, dout=12; in Layer 2, din=12, dout=6。

KANLayer:

The computational complexity is , where d = 6.

Fully Connected Layer：

The computational complexity is , where *d_in_* = 6, *d_out_* = 2.

The overall model complexity is primarily contributed by the two R-GCN layers, approximately .

| **Table S2.** The results of experiments on simulated data | | | | | |  |
| --- | --- | --- | --- | --- | --- | --- |
|  | Pbsv | Svim | cuteSV | Sniffles | MAMnet | GKNnet |
| Precision | 85.73% | 71.41% | 72.09% | 72.31% | 70.52% | 77.81% |
| Recall | 63.32% | 65.24% | 73.26% | 66.15% | 67.27% | 74.01% |
| F1 | 72.84% | 68.19% | 72.67% | 69.14% | 68.86% | 75.86% |
|  |  |  |  |  |  |  |

| **Table S3.** The performance comparison of SV callers on ONT dataset about ERR8562466 | | | | | |
| --- | --- | --- | --- | --- | --- |
|  | Pbsv | Svim | Sniffles | MAMnet | GKNnet |
| 210X |  |  |  |  |  |
| Precision | 49.72% | 55.56% | 41.21% | 42.17% | 48.04% |
| Recall | 45.92% | 30.61% | 37.68% | 35.29% | 52.49% |
| F1 | 47.75% | 39.47% | 39.37% | 38.42% | 50.17% |
| 168X |  |  |  |  |  |
| Precision | 48.65% | 45.11% | 40.17% | 41.57% | 47.56% |
| Recall | 45.23% | 29.08% | 30.65% | 34.26% | 51.05% |
| F1 | 46.88% | 35.36% | 34.77% | 37.56% | 49.24% |
| 126X |  |  |  |  |  |
| Precision | 48.85% | 44.64% | 37.85% | 39.45% | 46.29% |
| Recall | 43.81% | 27.69% | 32.08% | 32.86% | 49.89% |
| F1 | 46.19% | 34.18% | 34.73% | 35.85% | 48.02% |
| 50X |  |  |  |  |  |
| Precision | 41.18% | 39.24% | 32.28% | 36.49% | 44.80% |
| Recall | 35.62% | 20.07% | 24.75% | 29.67% | 46.89% |
| F1 | 38.20% | 26.52% | 28.02% | 32.73% | 45.82% |

| **Table S4.** The performance comparison of SV callers on PacBio dataset about DRR095880 | | | | | |
| --- | --- | --- | --- | --- | --- |
|  | Pbsv | Svim | Sniffles | MAMnet | GKNnet |
| 250X |  |  |  |  |  |
| Precision | 54.95% | 50.98% | 54.24% | 48.96% | 53.83% |
| Recall | 44.35% | 48.10% | 45.57% | 44.37% | 48.76% |
| F1 | 49.08% | 49.50% | 49.53% | 46.55% | 51.17% |
| 125X |  |  |  |  |  |
| Precision | 52.54% | 50.67% | 44.72% | 44.57% | 51.79% |
| Recall | 39.24% | 36.83% | 35.02% | 41.68% | 46.27% |
| F1 | 44.93% | 42.66% | 39.28% | 43.08% | 48.87% |
| 60X |  |  |  |  |  |
| Precision | 31.55% | 11.08% | 36.06% | 40.08% | 46.52% |
| Recall | 42.45% | 53.24% | 37.41% | 35.73% | 39.64% |
| F1 | 36.20% | 18.34% | 36.72% | 37.78% | 42.81% |
| 30X |  |  |  |  |  |
| Precision | 23.49% | 10.86% | 32.84% | 36.55% | 44.28% |
| Recall | 39.39% | 46.46% | 34.44% | 34.72% | 36.02% |
| F1 | 29.43% | 17.61% | 33.62% | 35.61% | 39.73% |

| **Table S5.** The performance comparison of the model and basel-ine methods across different SV length intervals | | | | | | |
| --- | --- | --- | --- | --- | --- | --- |
|  | Pbsv | Svim | cuteSV | Sniffles | MAMnet | GKNnet |
| 50-200 |  |  |  |  |  |  |
| Precision | 93.43% | 86.99% | 90.02% | 87.88% | 85.64% | 91.28% |
| Recall | 86.35% | 91.45% | 91.45% | 87.67% | 84.39% | 90.53% |
| F1 | 89.75% | 89.17% | 90.73% | 87.78% | 85.01% | 90.90% |
| 200-500 |  |  |  |  |  |  |
| Precision | 82.79% | 72.58% | 72.02% | 71.92% | 76.98% | 83.88% |
| Recall | 76.31% | 87.99% | 87.71% | 87.99% | 77.23% | 81.84% |
| F1 | 79.42% | 79.55% | 79.09% | 79.15% | 77.10% | 82.85% |
| 500-1000 |  |  |  |  |  |  |
| Precision | 84.44% | 27.36% | 28.36% | 28.15% | 78.31% | 84.91% |
| Recall | 62.68% | 90.68% | 92.68% | 91.35% | 82.07% | 90.24% |
| F1 | 71.95% | 42.04% | 43.43% | 43.04% | 80.15% | 87.49% |
| 1000-5000 |  |  |  |  |  |  |
| Precision | 81.60% | 37.67% | 38.36% | 38.08% | 57.33% | 62.20% |
| Recall | 52.86% | 80.71% | 82.14% | 80.71% | 70.26% | 77.86% |
| F1 | 64.16% | 51.36% | 52.30% | 51.75% | 63.14% | 69.16% |
| 5000- |  |  |  |  |  |  |
| Precision | 85.01% | 87.98% | 89.16% | 88.75% | 65.39% | 69.85% |
| Recall | 55.03% | 51.95% | 58.16% | 50.34% | 67.47% | 71.17% |
| F1 | 66.81% | 65.53% | 70.40% | 64.25% | 66.41% | 70.50% |

**Table S6.** The performance comparison of SV callers on Kluyveromyces lactis dataset about ERR12454982

|  | Pbsv | Sniffles | Svim | GKNnet |
| --- | --- | --- | --- | --- |
| 470X |  |  |  |  |
| Precision | 79.49% | 72.48% | 82.63% | 78.61% |
| F1 | 69.22% | 66.47% | 56.11% | 75.94% |
| Recall | 74% | 69.35% | 66.84% | 77.25% |
| 235X |  |  |  |  |
| Precision | 70.4% | 67.48% | 71.3% | 72.39% |
| F1 | 61.27% | 59.7% | 52.64% | 69.66% |
| Recall | 65.52% | 63.35% | 60.57% | 71% |
| 117X |  |  |  |  |
| Precision | 61.46% | 59.65% | 62.65% | 64.79% |
| F1 | 53.23% | 51.02% | 51.59% | 60.46% |
| Recall | 57.05% | 55% | 56.58% | 62.55% |
| 58X |  |  |  |  |
| Precision | 53.98% | 50.84% | 54.39% | 55.84% |
| Recall | 45.01% | 44.3% | 46.09% | 52.39% |
| F1 | 49.09% | 47.35% | 49.9% | 54.06% |

**Table S7.** The performance comparison of SV callers on Penicillium dataset about SRR17178875

|  | Pbsv | Sniffles | Svim | GKNnet |
| --- | --- | --- | --- | --- |
| 150X |  |  |  |  |
| Precision | 78.06% | 81.76% | 85.38% | 83.78% |
| Recall | 53.3% | 67.74% | 63.55% | 72.62% |
| F1 | 63.55% | 74.89% | 72.87% | 77.8% |
| 75X |  |  |  |  |
| Precision | 67.82% | 73.44% | 74.89% | 75.13% |
| Recall | 53.67% | 64.84% | 60.31% | 66.58% |
| F1 | 59.92% | 68.87% | 66.81% | 70.6% |
| 39X |  |  |  |  |
| Precision | 59.58% | 63.76% | 67.35% | 66.94% |
| Recall | 47.2% | 55.71% | 55.88% | 59.23% |
| F1 | 52.67% | 59.46% | 61.08% | 62.85% |

**Performance of running time and memory usage**

To evaluate the runtime, CPU time, and memory usage of each variant caller, we conducted tests using a simulated dataset on the following hardware specifications: CPU: Intel i9-10980XE (18 cores, 36 threads, 3.0 GHz base frequency), GPU: NVIDIA GeForce RTX 4090. The test results are shown in Table S5.The results indicate that cuteSV, Svim, and Sniffles have similar runtimes, each taking about 10 minutes. In contrast, Pbsv takes longer, approximately 30 minutes, GKNnet has the longest runtime, taking about 40 minutes. This difference is primarily due to GKNnet’s deep learning model, which involves more complex computation processes requiring greater computational resources and time, resulting in longer runtimes.Regarding CPU usage, almost all methods fully utilized 36 threads, indicating that these tools efficiently allocate computational resources during processing. However, despite the longer runtime of GKNnet, its memory usage is relatively low, demonstrating good memory management capabilities. This is mainly due to our approach of splitting the entire dataset into smaller parts and processing them in batches, which reduces memory consumption.Although GKNnet does not perform as well in terms of runtime efficiency, its more complex computation process provides superior performance in variant detection compared to traditional methods. GKNnet offers significant advantages in accuracy and stability, meaning that despite its longer runtime, it delivers more reliable and precise results when handling complex variant detection tasks.

**Table S8.** The Performance Comparison of Variant Callers

| Metric | cuteSV | Pbsv | Svim | Sniffles | GKNnet |
| --- | --- | --- | --- | --- | --- |
| Elapsed Time | 589 | 1951 | 654 | 678 | 2376 |
| CPU Time | 13742 | 35257 | 14952 | 16737 | 42478 |
| Maximum Memory Usage | 1276M | 4278M | 3158M | 3486M | 958M |
